# A new paradigm for applying deep learning to protein–ligand interaction prediction

**DOI:** 10.1093/bib/bbae145

**Published:** 2024-04-05

**Authors:** Zechen Wang, Sheng Wang, Yangyang Li, Jingjing Guo, Yanjie Wei, Yuguang Mu, Liangzhen Zheng, Weifeng Li

**Affiliations:** School of Physics, Shandong University, South Shanda Road, 250100 Shandong, China; Shanghai Zelixir Biotech, Xiangke Road, 200030, Shanghai, China; School of Physics, Shandong University, South Shanda Road, 250100 Shandong, China; Centre in Artificial Intelligence Driven Drug Discovery, Faculty of Applied Sciences, Macao Polytechnic University, Rua de Luís Gonzaga Gomes, Macao, China; Shenzhen Institute of Advanced Technology, Chinese Academy of Sciences, Xueyuan Road 1068, Shenzhen, 518055 Guang Dong, China; School of Biological Sciences, Nanyang Technological University, Singapore; Shanghai Zelixir Biotech, Xiangke Road, 200030, Shanghai, China; Shenzhen Institute of Advanced Technology, Chinese Academy of Sciences, Xueyuan Road 1068, Shenzhen, 518055 Guang Dong, China; School of Physics, Shandong University, South Shanda Road, 250100 Shandong, China

**Keywords:** protein–ligand interaction, scoring function, deep learning, graph neural network

## Abstract

Protein–ligand interaction prediction presents a significant challenge in drug design. Numerous machine learning and deep learning (DL) models have been developed to accurately identify docking poses of ligands and active compounds against specific targets. However, current models often suffer from inadequate accuracy or lack practical physical significance in their scoring systems. In this research paper, we introduce IGModel, a novel approach that utilizes the geometric information of protein–ligand complexes as input for predicting the root mean square deviation of docking poses and the binding strength (pKd, the negative value of the logarithm of binding affinity) within the same prediction framework. This ensures that the output scores carry intuitive meaning. We extensively evaluate the performance of IGModel on various docking power test sets, including the CASF-2016 benchmark, PDBbind-CrossDocked-Core and DISCO set, consistently achieving state-of-the-art accuracies. Furthermore, we assess IGModel’s generalizability and robustness by evaluating it on unbiased test sets and sets containing target structures generated by AlphaFold2. The exceptional performance of IGModel on these sets demonstrates its efficacy. Additionally, we visualize the latent space of protein–ligand interactions encoded by IGModel and conduct interpretability analysis, providing valuable insights. This study presents a novel framework for DL-based prediction of protein–ligand interactions, contributing to the advancement of this field. The IGModel is available at GitHub repository https://github.com/zchwang/IGModel.

## INTRODUCTION

Understanding the precise binding poses of ligands within protein receptor structures is of immense significance in drug design [1–4]. Accurately predicting ligand binding poses provides valuable insights into the intricate mechanisms underlying drug–target interactions. This knowledge enables researchers to optimize binding affinity, selectivity and pharmacological properties, ultimately leading to the development of more potent and efficient therapeutic agents [5, 6].

However, experimental methods for determining the binding mode of small molecules within proteins are often inefficient and their accuracy is not always guaranteed [7, 8]. Experimental techniques, such as X-ray diffraction offers atomic insights into protein–ligand interactions but are often time-consuming and resource-intensive. They may also face challenges in obtaining high-resolution structures or capturing the dynamic aspects of the binding process [9, 10]. Furthermore, experimental approaches may be constrained by the availability of suitable protein samples and the complexity of the system under investigation. Therefore, relying solely on experimental techniques for determining ligand binding poses may not meet the demands of high-throughput drug discovery and design [11].

Computational methods, such as molecular docking, play a crucial role in this endeavor by offering efficient and accurate tools to explore the vast chemical space and guide rational drug design efforts [12–15]. Scoring functions (SFs) are developed to guide fast and accurate prediction of protein–ligand complex structures, particularly the ligand binding pattern when the protein structure is already known or determined [16, 17]. SFs calculate the binding energy or score associated with each pose, allowing for the ranking and selection of the most favorable binding configurations [9, 17]. These SFs consider various factors, including molecular shape complementarity, electrostatic interactions, van der Waals forces, hydrogen bonding and solvation effects [11], to estimate the overall binding affinity or likelihood of a ligand pose being biologically relevant. SFs facilitate the exploration of ligand conformational space by identifying potential binding modes and providing insights into the strength and specificity of ligand–receptor interactions [18]. They allow researchers to prioritize ligand poses for further analysis or optimization, thereby aiding in the rational design of drug candidates with enhanced binding affinity and selectivity [9, 19].

In the 1980s, rules were designed based on physical knowledge and expert experience, or by using force field parameters from bio-molecule simulation systems, which formed the early SFs [20]. However, traditional SFs, usually polynomials with few parameters and atom-type definitions, are not accurate enough to describe the conformational space of protein–ligand complexes [21]. Another category of SFs involves machine learning (ML)-based approaches, which have been developed since 2004 [22]. These SFs predict ligand binding affinity by extracting interaction features using various ML techniques [23, 24]. Notable SFs, such as RF-Score [25] (including its virtual screen version, RF-score-VS), NN-Score [26], AGL-Score [27], have achieved excellent performance (‘scoring power’; [28]) in PDBbind-related benchmarks. With the availability of public experimental data and advancements in deep learning (DL) algorithms, there has been a rising trend of utilizing DL models for predicting protein–ligand interactions [29–31]. Since 2017, various DL-based models have emerged for predicting protein–ligand affinity. AtomNet [32], K$_{deep}$ [33] and Pafnucy [34] have utilized 3D convolutional neural networks (CNNs) to correlate the three-dimensional mesh representation of a complex with its affinity. Additionally, our group has developed two models based on 2D convolution—OnionNet and OnionNet-2—which estimate protein–ligand affinity by tallying the number of contacts between ligand atoms and protein atoms/residues across various distance intervals [35, 36]. Nevertheless, recent studies indicate that ML or DL models trained solely on native structures tend to underperform in tasks related to docking and screening power, despite their robust performance in scoring power [37]. To make the models applicable to practical scenarios, researchers have designed predictors that can directly identify near-native poses of the ligand or screen active compounds. For instance, DeepBSP uses 3D CNN to predict the root mean square deviation (RMSD) of ligand docking poses [38]. DeepRMSD+Vina, previously proposed by our group, adopts modified formats of van der Waals and Coulombic terms used in traditional force fields as features and has been effective in docking power and docking pose optimization [39]. Additionally, DeepDock utilizes a graph neural network to learn the distance probability distribution between protein–ligand atoms rather than directly predicting the protein–ligand binding affinity or ligand pose RMSD. [40]. Building upon the idea of DeepDock, RTMScore introduces a novel graph representation and employs a graph transformer model to learn distance probability distributions, achieving state-of-the-art (SOTA) results across multiple virtual screening test sets and docking pose prediction tasks [41]. However, it has been observed that many DL SFs perform poorly for all evaluation metrics [28]. More researchers now recognize the importance of achieving balanced performance for both docking and screening tasks [42–44]. Among the numerous DL methods mentioned above, some provide affinity prediction or ligand pose prediction, but few can offer directly interpretable indicators with physical meanings, which is more intuitive for computational drug developers [28, 45]. Other methods lack robustness, especially when using predicted structures or cross-docked structures as protein templates. There are few approaches that can accurately predict the optimal ligand binding poses [46, 47].

To address these limitations, we propose an SF based on the geometric graph neural network, IGModel, to enhance the prediction of protein–ligand interactions, especially the docking poses prediction. The input of IGModel consists of two graphs: one represents the protein binding pocket, and the other represents the protein–ligand atomic interactions. Unlike previous graph representations [48, 49], IGModel incorporates the distance and orientation between interacting atoms as geometric features of the complex, providing a more comprehensive description of the relative positions of the ligand within the binding pocket. We employ EdgeGAT layers proposed by Kamiński et al. [50] to encode protein–ligand interactions and derive a latent vector, which is subsequently decoded to yield the RMS) of the pose and the binding strength to the protein via two decoding modules. Hence, IGModel can be segmented into two sub-branches: IGModel$_{RMSD}$ and IGModel$_{pkd}$. In the CASF-2016 [28] test for docking power, IGModel$_{RMSD}$ attained the highest Top1 docking success rate (97.5% with native poses included and 95.1% when excluded). Additionally, IGModel has shown commendable performance on cross-docking datasets such as the PDBbind-CrossDocked-Core set [51] and the DISCO [52], comparable to or even surpassing other baseline models. Additionally, IGModel demonstrates excellent performance on unbiased test sets and datasets containing target structures generated by AlphaFold2 [53], proving its outstanding generalizability and practicality for drug discovery. The model captures key charge-charge or hydrogen bond interactions, indicating potential robustness for protein–ligand interaction prediction and suggesting its potential use for lead optimization. Overall, we present a highly accurate ligand pose and binding affinity prediction model with enhanced drug design capability.

## METHODS

### Datasets

In this study, we utilized the native protein–ligand complexes from the PDBbind database (v.2019) [54] and their corresponding docking poses for training. To increase the diversity of ligand binding conformations, an average of 15 docking poses were generated for each native protein–ligand complex using AutoDock Vina [55] and ledock [56]. We excluded protein–ligand pairs from the CASF-2016 test set and those with peptide ligands, as well as any samples that could not be parsed by certain programs, such as rdkit [57]. The actual RMSD of the docking poses was calculated using the spyrmsd package [58]. The binding affinity (pKd) of the native protein–ligand complex is expressed as the negative logarithm of the dissociation constant (K$_{d}$), the inhibition constant (K$_{i}$) or the half-inhibitory concentration (IC50). The relationship between binding free energy and K$_{d}$ is $\Delta $G = RT*ln*K$_{d}$, where R is the gas constant and T is the temperature. At the room temperature, *T* = 298.15 K, $\Delta $*G* = −1.3633 pKd. The division of the training and validation sets follows the same procedure as in previous work [39], wherein 1000 protein–ligand pairs were randomly selected from the refine set as the validation set, and all remaining samples from the general set were used as the training set. This ensures that there are no overlapping protein–ligand pairs in training, validation and test sets. The parameters used for molecular docking and the distributions of RMSD and pKd for docking poses are shown in Supplementary part 1.

### Graphical representation of protein–ligand complexes

Generally, the binding of a ligand within the pocket is governed by non-covalent interactions, and the potential energy surfaces associated with protein–ligand binding disclose preferred geometries among atoms. Therefore, to accurately describe the relative positions of atoms in the protein–ligand system, which is crucial for characterizing protein–ligand interactions, we construct a heterogeneous graph G$^{RR, LL, RL}$. This graph consists of two types of nodes: protein atom nodes (*V*$^{R}$) and ligand atom nodes (*V*$^{L}$). To conserve computational resources, only protein atoms within 8 Å of the ligand co-crystal structure are used for interaction modeling. There are four message-passing channels in G$^{RR, LL, RL}$, namely *E*$^{RR}$ (*V*$^{R}$ to *V*$^{R}$), *E*$^{LL}$ (*V*$^{L}$ to *V*$^{L}$), *E*$^{RL}$ (*V*$^{R}$ to *V*$^{L}$) and *E*$^{LR}$ (*V*$^{L}$ to *V*$^{R}$).

**Figure 1:**
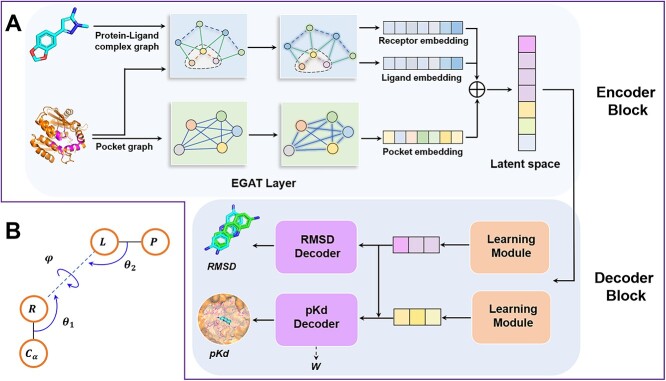
Overview of IGModel. (**A**) Illustration of predicting RMSD and pKd from the protein–ligand complex structure. (**B**) The orientation information on the relative positions of protein–ligand atoms. C$_{\alpha }$, R, L and P represent the C$_{\alpha }$ of the residue, a certain atom within the residue, a certain atom within the ligand and the geometric center of the ligand, respectively. The $\theta _{1}$ and $\theta _{2}$ are the angles between C$_{\alpha }$-R and L-R, and between P-L and R-L, respectively. The $\psi $ denotes the dihedral angle formed by C$_{\alpha }$, R, L and P.

Within the ligand subgraph G$^{L}$=(*V*$^{L}$,*E*$^{LL}$), we define seven types of ligand atoms: C, N, O, P, S, Hal (representing halogen F, Cl, Br, and I), and DU (representing other element types). If a covalent bond exists between atoms *i* and *j*, an edge *e*$_{ij}^{L}$ is defined between nodes *v*$_{i}^{L}$ and *v*$_{j}^{L}$. The rdkit package enables the extraction of physical and chemical information pertaining to chemical bonds, which serves as the edge feature alongside bond length. For the protein subgraph G$^{R}$=(*V*$^{R}$,*E*$^{RR}$), we redefine atom types based on the element type, the residue type to which they blong, and their location in either the main chain or the side chain. For example, ‘LYS-MC’ and ‘LYS-SC’ represent the carbon atom on the main chain and side chain of the lysine residue, respectively. The one-hot encoding of atom types, combined with aromaticity, charge, and distance to the $\alpha $-C atom, are collectively used as node features for G$^{R}$. When the distance between two nodes *v*$_{i}^{R}$ and *v*$_{j}^{R}$ is less than 5 Å, an edge *e*$_{ij}^{R}$ is established, and the edge length serves as the edge feature. If the distance between a protein node *v*$_{i}^{R}$ and a ligand node *v*$_{i}^{L}$ is less than 8 Å, directed edges *e*$_{i,i}^{RL}$ and *e*$_{i,i}^{LR}$ are created, where *e*$_{i,i}^{RL}$ denotes the direction from *v*$_{i}^{R}$ to *v*$_{i}^{L}$, and *e*$_{i,i}^{LR}$ represents the direction from *v*$_{i}^{L}$ to *v*$_{i}^{R}$. This multilateral architecture facilitates the integration of node and edge information within the protein–ligand graph. To provide a more comprehensive description of the relative positions between protein–ligand atoms, in addition to considering the interatomic distance, we introduce a novel orientation feature. Specifically, we introduce a dihedral angle $\varphi $ (formed by the geometric center of the pose, *v*$_{i}^{L}$, *v*$_{i}^{R}$ and $\alpha $-C), as well as two angles $\theta _{1}$ and $\theta _{2}$, as shown in Figure 1(B). In Figure 1(B), C$_{\alpha }$, R, L and P represent the C$_{\alpha }$ of the residue, a specific atom within the residue, a specific atom within the ligand and the geometric center of the ligand, respectively. $\theta _{1}$ and $\theta _{2}$ are the angles formed between the C$\alpha $-R edge and the L-R edge, as well as the P-L edge and R-L edge, respectively. Finally, $\sin $($\varphi $/2), $\cos $($\theta _{1}$/2), $\cos $($\theta _{2}$/2) and distance are used as features for the interaction edge. Table S1 summarizes all the node and edge features within this heterogeneous graph.

The physicochemical environment of the binding pocket is critical for ligand binding [59, 60]. However, macroscopic descriptions of binding pockets cannot be adequately represented solely by atomic-level interaction graphs. To overcome this challenge, we construct an undirected graph G$^{p}$=(*V*$^{p}$, *E*$^{p}$) to characterize the residue states in the binding pocket. In a manner akin to RTMScore, we define the binding pocket as the residues within 8Å of the co-crystallized ligand. Each node *v*$_{i}^{p}\in $*V*$^{p}$ represents a residue within the binding pocket, and an edge *e*$_{ij}^{p}$ is established when the minimum distance between two residues is less than 10 Å. Node features primarily include the residue type, distance distribution between internal atoms, and the position relative to the pocket’s center. Meanwhile, edge features convey the distances between nodes associated with the main chain atoms. Table S2 provides a comprehensive summary of the detailed node and edge features of the binding pocket graph.

### Architecture

IGModel comprises three main components: the interaction feature encoding module, the RMSD decoding module and the pKd decoding module.

The encoding portion of IGModel includes two branches that are tasked with processing the binding pocket graph and the protein–ligand interaction graph, respectively, with each branch featuring two EdgeGAT layers. When the binding pocket graph is input into the encoder, its node features h$_{pock}$ and edge features f$_{pock}$ are updated as shown in Equation (1). The updated node features h$^{\prime }_{pock}$ are converted into a vector of length 1024 to represent the binding pocket embedding: 


(1) \begin{align*}& h^{\prime}_{pock}, f^{\prime}_{pock} = EGATConv(EGATConv(h_{pock}, f_{pock}))\end{align*}

For the protein–ligand interaction graph, the initial message-passing phase is shown in Equations (2)–(5). Following this phase, the nodes representing the protein and ligand atoms are updated. The revised nodes of the protein and ligand subgraphs, denoted as h$^{\prime }_{rec}$ and h$^{\prime }_{lig}$, are as shown in Equations (6)–(7). After undergoing two rounds of updates, the node features for both the protein and the ligand are converted into 1024-dimensional vectors, which acts as embeddings encapsulating details about the protein and ligand atoms. The amalgamation of these two embeddings, along with the binding pocket embedding, constitutes the final latent space that encapsulates the entirety of the protein–ligand interaction: 


(2) \begin{align*} h^{\prime}_{rec1}, f^{\prime}_{rec1} = EGATConv(h_{rec}, f_{rec})&& \end{align*}

(3) \begin{align*} h^{\prime}_{rec2}, f^{\prime}_{lig-rec} = EGATConv((h_{lig}, h_{rec}), f_{lig-rec}) \end{align*}

(4) \begin{align*} h^{\prime}_{lig1}, f^{\prime}_{lig1} = EGATConv(h_{lig}, f_{lig})&& \end{align*}

(5) \begin{align*} h^{\prime}_{lig2}, f^{\prime}_{rec-lig} = EGATConv((h_{rec}, h_{lig}), f_{rec-lig})& \end{align*}

(6) \begin{align*} h^{\prime}_{rec} = BatchNorm(h^{\prime}_{rec1}) + BatchNorm(Linear(h^{\prime}_{rec2})) \end{align*}

(7) \begin{align*} h^{\prime}_{lig} = BatchNorm(h^{\prime}_{lig1}) + BatchNorm(Linear(h^{\prime}_{lig2}))\end{align*}

Each learning module in the decoding part consists of a gMLP [61] layer and two linear layers. After passing through the two learning modules, the latent space is converted into two 128-dimensional vectors, *V*$_{RMSD}$ and *V*$_{pkd}$. To ensure that the predicted pKd considers the change in RMSD of the ligand pose, we map *V*$_{RMSD}$ to a new vector *V*$^{\prime }_{pkd}$ in the space of *V*$_{pkd}$ through a linear layer. This new vector is integrated with *V*$_{pkd}$ for decoding the pKd.

The training dataset encompasses both experimentally verified structures and virtual conformations produced through molecular docking. However, binding affinity data is solely available for native complexes. It is postulated that the binding strength of a given pose to its target is inversely proportional to its RMSD from the native conformation, with the native protein–ligand conformation processing the highest binding affinity. This study aims to decode the relationship between the RMSD of docking poses and their binding strength using DL. Additionally, the model is designed to predict a decay factor $W$ (illustrated in Figure 1A and defined in Equation 8) that ranges from of 0 to 1 within the pKd decoding segment, to portray the diminishing pKd value as the RMSD increases. Using $W$ and the previous assumptions, the binding strength (pKd$_{label}$) of the docking pose to the receptor can be deduced, which serves as the label for pKd (Equation 9): 


(8) \begin{align*} & W = Sigmoid(Linear(V^{\prime}_{pkd} + V_{pkd})) \end{align*}

(9) \begin{align*} & pKd_{label} = pKd_{nat} - W * RMSD_{real}\end{align*}

In Equation (8), pKd$_{nat}$ represents the binding affinity between the native conformation of the ligand and the receptor, and RMSD$_{real}$ is the real RMSD of the docking pose. When RMSD$_{real}$ is close to 0, pKd$_{label}$ will be close to pKd$_{nat}$.

The loss function during training is defined as follows: 


(10) \begin{align*}& \begin{split} \mathcal{L} = \alpha * mse(RMSD_{real}, RMSD_{pred}) + \\ \beta * mse(pKd_{label}, pKd_{pred}) + \gamma * \frac{1}{N} \sum pKd_{pred} \end{split}\end{align*}

where weights $\alpha $, $\beta $, and $\gamma $ are used to sum up the components within the loss function, which are set as 1, 0.5 and 0.05 in this study. N is the number of samples in a mini-batch. According to our assumption, the binding strength between the ligand and the target exhibits a negative correlation with the RMSD of the ligand binding pose. The last term in the loss function is designed to ensure that IGModel fits pKd in a ‘descending’ direction, facilitating a negative correlation between the predicted pKd and the RMSD.

## RESULTS

### Evaluation on CASF-2016 benchmark

**Figure 2:**
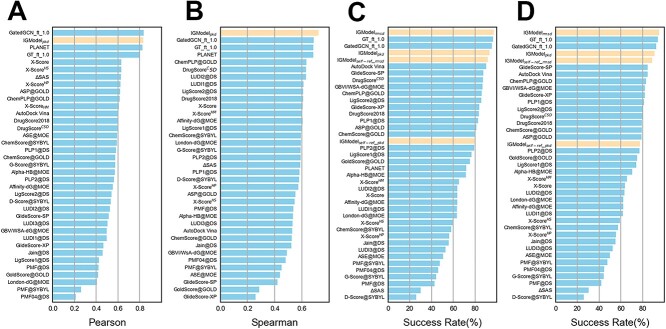
Comparison of scoring, ranking, and docking power using the CASF-2016 benchmark against other SFs. (**A**) Scoring power measures the correlation between the scores of the model and experimental affinity, using the PCC as the metric. (**B**) Ranking power evaluates the ability of a SF to rank the known ligands for a certain target, with the Spearman’s correlation coefficient serving as the metric. (**C**) and (**D**) display the top 1 success rate when the crystal structures are included and excluded from the test set, respectively.

The performance of the SF is evaluated across four dimensions: scoring power, ranking power, docking power, and screening power, as defined by the CASF-2016 benchmark [28]. Previous research indicates that SFs with robust docking or screening power often exhibit weaker scoring and ranking power, such as DeepRMSD+Vina [39] and RTMScore [41]. Our model aims to achieve balanced performance across various tasks. Despite primarily focusing on identifying near-native conformations of the ligand (docking power), what is surprising is that IGModel also demonstrates a relatively balanced performance in other tasks (Figure 2 and Tables S3–S4). Firstly, for docking power, the top 1 success rate of IGModel$_{rmsd}$ with native poses included in the test set is 97.5%, and the value remains as high as 95.1% when the native poses are excluded. At the same time, IGModel$_{pkd}$ also achieves higher top 1 success rates compared to most models, which are 93.3 and 90.9$\%$ when crystal structures are included and excluded in the test set, respectively. The ablation experiments of IGModel on the validation set and the CASF-2016 docking power are detailed in Supplementary part 4. We also conducted a ‘binding funnel analysis’ by calculating the Spearman’s correlation coefficient (SCC) between the RMSD or pKd predicted by the IGModel and the actual RMSD for the decoy set of each protein–ligand pair, and then calculating the average SCC across different RMSD intervals. The result indicates that IGModel maintains a clear advantage across various RMSD ranges (Figure S2). However, the cocrystallized ligand may be unknown in actual virtual screening, thus we retrain the the IGModel (called IGModel$_{self-ref}$) using the docking poses themselves as the reference to determine the binding pocket of the target. In this scenario, the performance of IGModel$_{self-ref}$ in the CASF-2016 docking power test significantly declined compared to the baseline version of IGModel (Table S3). Recently, AI-based protein–ligand blind docking algorithms have been significatly developed, such as DiffDock, a generative model based on the diffusion model [62]. We attempted to regenerate the binding poses of protein–ligand complex from the CASF-2016 test set using DiffDock. Subsequently, these binding poses were scored by IGModel, GatedCGN${}\underline {\ \ }$ft${}\underline {\ \ }$1.0, GT${}\underline {\ \ }$ft${}\underline {\ \ }$1.0 and Vina. By analyzing the PCC and SCC values obtained from these SFs, we further demonstrated the versatility of IGModel (see detail in Supplementary part 5).

**Figure 3:**
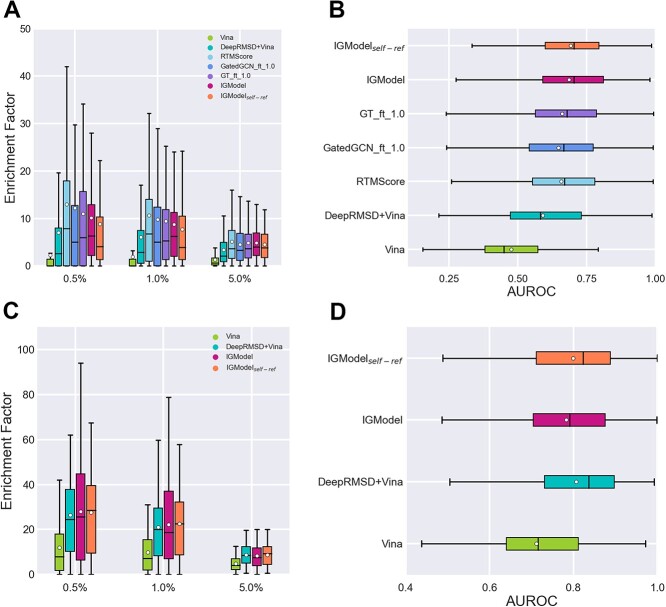
The performance of SFs on the DUD-E and DUD-AD sets. (**A**) and (**C**) show the EF (0.5, 1.05 and 5.0%) achieved by the SFs on the DUD-AD and DUD-E datasets, respectively. (**B**) and (**D**) present the AUROC of SFs on the DUD-AD and DUD-E datasets, respectively. The white circles in the boxplot denotes the mean value within each group of data. The boxplot for RTMScore and GenScore on DUD-E set can be found on the original papers.

Additionally, screening power refers to the ability of the SFs to identify the true binders to a specific receptor among a large library of compounds. This is measured by two indicators: the first is the success rate of identifying the highest-affinity binder among the top 1, 5 or 10$\%$ of ranked ligands across all 57 target proteins in the test set; the second is the enrichment factor (EF), which is calculated by the average percentage of true binders among the top 1, 5 or 10$\%$ of ranked candidates across all 57 targets. IGModel achieves a top 1% success rate of 66.7%, which is comparable to RTMScore [41], but slightly lower than GT${}\underline {\ \ }$ft${}\underline {\ \ }$1.0 [43]. However, the EF achieved by IGModel$_{pkd}$ is only 19.4, which is significantly lower than that of RTMScore and GT${}\underline {\ \ }$ft${}\underline {\ \ }$1.0, but still higher than most predictors, such as DeepDock [40]. In general, IGModel exhibits a relatively balanced performance across various metrics based on the CASF-2016 benchmark. Interestingly, for scoring power, IGModel$_{pkd}$ achieved a Person correlation coefficient (PCC) of 0.831, very close to the 0.834 and 0.824 achieved by GatedCGN${}\underline {\ \ }$ft${}\underline {\ \ }$1.0 [43] and PLANET [42], respectively. The scatter plots of IGModel$_{pkd}$ on the validation set and CASF-2016 core set are shown in Figure S3. Finally, for the ranking power test, IGModel$_{pkd}$ achieved a SCC of 0.723, higher than the 0.686 achieved by GatedCGN${}\underline {\ \ }$ft${}\underline {\ \ }$1.0 (Table S4). To further assess the screening power of IGModel, the DUD-E and DUD-AD sets were utilizes as additional test sets. On the DUD-E set, RTMScore, GatedCGN${}\underline {\ \ }$ft${}\underline {\ \ }$1.0 and GT${}\underline {\ \ }$ft${}\underline {\ \ }$1.0 demonstrate a significant advantage over IGModel, both in terms of EF and AUROC. On the unbiased DUD-AD set, RTMScore, GatedCGN${}\underline {\ \ }$ft${}\underline {\ \ }$1.0 and GT${}\underline {\ \ }$ft${}\underline {\ \ }$1.0 still exhibited optimal EF, despite their AUROC being slight lower than that of IGModel. This indicates that there is still a gap between IGModel and SOTA SFs in screening tasks. The detailed results are presented in Figure 3 and Supplementary Part 6.

### Evaluation on the redocking and cross-docking test sets

Currently, in most molecular docking applications, the protein is treated as a rigid molecule, which deviates from the actual protein–ligand binding behavior observed in reality. This is because the binding pocket can accommodate different compounds with flexible side chains and, sometimes, even with adjustable backbones [63]. Redocking and cross-docking are two methods for evaluating molecular docking. Redocking involves reintroducing a ligand into its cognate receptor, while cross-docking pertains to docking a ligand into a non-cognate receptor within the original pocket [52]. To comprehensively assess the docking power of IGModel, various redocking and cross-docking benchmarks have been adopted.

**Figure 4:**
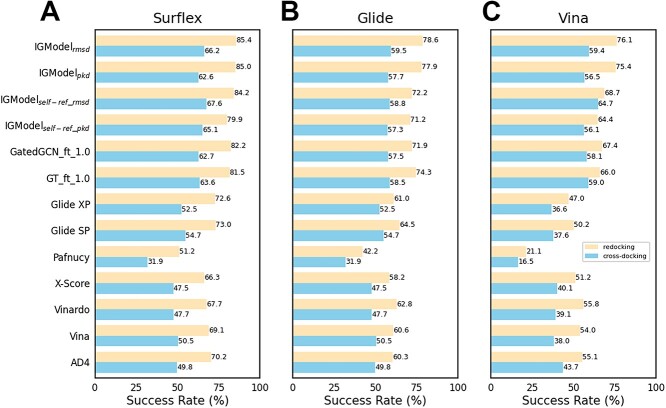
Docking power of IGModel and other SFs on PDBbind-CrossDocked-Core set. (**A**), (**B**) and (**C**) show the top 1 success rate of SFs on poses generated by Surflex, GLide and AutoDock Vina, respectively. The results of SFs other than IGModel are cited from the reference.

The first test set we assessed is PDBbind-CrossDocked-Core [51], with all receptors and ligands derived from the PDBbind v2016 core set. Each ligand was extracted from 285 protein–ligand crystal structures, and then was redocked into the original protein or four other proteins belonging to the same target cluster using three docking software: Surflex-Dock, Glide SP and AutoDock Vina. IGModel was tested in these three groups of poses and compared with other SFs. The results are shown in Figure 4. For cross-docking, the top 1 success rates on poses generated by IGModel$_{rmsd}$ with Surflex, Glide and Vina are 0.662, 0.595 and 0.594, respectively, slightly better than GT${}\underline {\ \ }$ft${}\underline {\ \ }$1.0 and GatedGCN${}\underline {\ \ }$ft${}\underline {\ \ }$1.0, but outperformed most predictors. Meanwhile, IGModel$_{pkd}$ still performed well, comparable to GatedGCN${}\underline {\ \ }$ft${}\underline {\ \ }$1.0, although it is slightly worse than the IGModel$_{rmsd}$. For redocking tasks, the top 1 success rates of IGModel$_{rmsd}$ and IGModel$_{pkd}$ on poses generated by Surflex, Glide and Vina are 0.854 and 0.850, 0.786 and 0.779, as well as 0.761 and 0.754, respectively, demonstrating a significant advantage over other SFs. However, it must be acknowledged that only few proteins have known native binding ligands and predicting protein pockets through computational methods remains a challenge, and this will restrict the applicability of SFs such as RTMScore, GenScore and IGModel, which define pockets based on the co-crystal ligand. Therefore, grabbing pockets through docking pose itself is suitable for all application scenarios. The overall performance of ${\text IGModel}_{self-ref\_{rmsd}}$ on the three cross-docking test sets is slightly superior to that of IGModel, although its performance on the redocking test sets is slightly worse than that of IGModel. This substantiates the practical application value of ${\text IGModel}_{self-ref\_{rmsd}}$ in real molecular docking scenarios.

**Figure 5:**
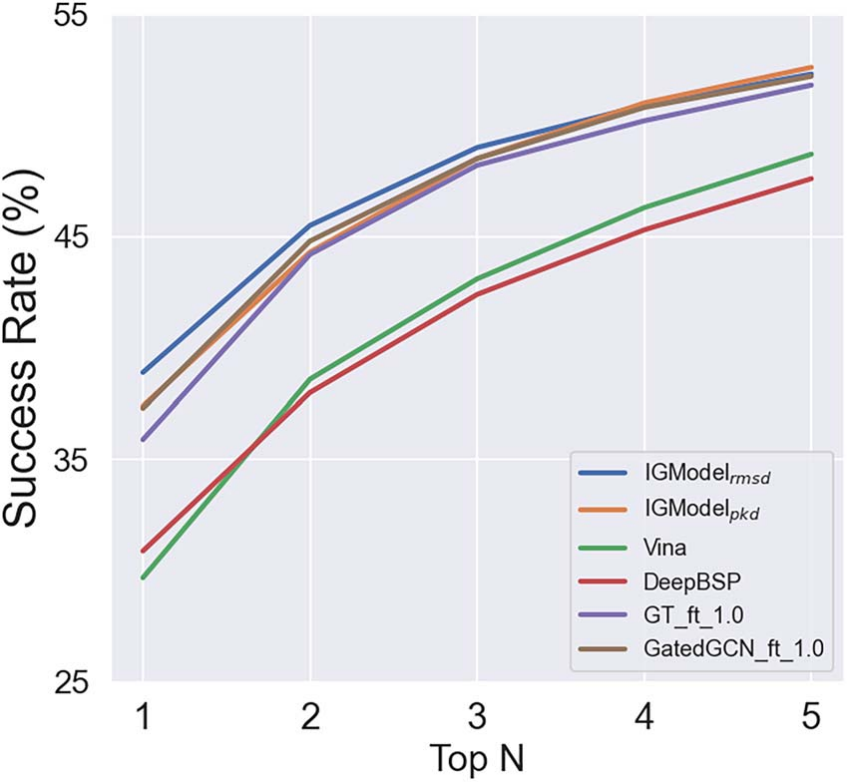
The top N success rate of IGModel and other baseline models on DISCO set.

Another cross-docking test set used in this study is DISCO, which contains 4399 crystal protein–ligand complexes across 95 protein targets [52]. The targets were derived from the DUD-E database [64], thereby encompassing a broad spectrum of protein families. Ligand poses were generated using AutoDock Vina [55], producing 20 poses per protein–ligand pair by default. For consistency with our prior research, we treated each target-ligand pair as an individual case when calculating the top 1 success rate, a method that differs from the integrated when tested on PDBbind-CrossDocked-Core set. The performance of IGModel in comparison to several baseline SFs on the DISCO set is depicted in Figure 5. It is evident that IGModel$_{rmsd}$ outperforms other baseline SFs, while IGModel$_{pkd}$ shows comparable results to GatedGCN${}\underline {\ \ }$ft${}\underline {\ \ }$1.0. The outstanding performance of IGModel in both redocking and cross-docking tasks underscores its significant practical value for applications.

### Generalization assessment of IGModel

Recent studies have shown that the performance of DL models is susceptible to biases within the data set, such as protein sequence similarity [65]. In this paper, we assessed the generalizability of the IGModel from two additional perspectives. Firstly, most SFs do not rigorously eliminate redundancy in the training set, which may result in better performance on the test set than in actual scenarios. To address this, we introduced an unbiased test set (the unbias-v2019 set in this work) that we previously proposed. This set contains protein–ligand pairs with low similarity to those in the PDBbind database v2019, and the native conformation of the ligands was excluded [46]. The similarity is determined by the product of protein sequence similarity (calculated by NW-align, http://zhanglab.dcmb.med.umich.edu/NW-align) and the Tanimoto similarity of the ligand Morgan fingerprints calculated by rdkit [57]. Secondly, predicting the binding pose of a ligand becomes even more challenging when the native conformation of the target is unknown. Computational methods such as AlphaFold2 [53] provide solutions for rapidly obtaining high-precision protein structures. Based on these predicted protein structures, molecular docking was applied to generate poses of the ligands. In our previous study, CASF-2016 and the unbias-v2019 sets were used to construct the test sets for the AlphaFold2 version, namely CASF-2016-AF2 and unbias-2019-AF2, respectively [46].

We evaluated the docking performance of SFs on the three mentioned datasets. Table 1 presents the Pearson correlation coefficient (PCC) and Spearman correlation coefficient (SCC) between the scores of SFs and the true RMSD. It is evident that IGModel demonstrates significantly higher accuracy compared to other baseline SFs. Particularly, on the unbias-v2019-AF2 set, IGModel maintains robust performance. The top 1 success rate achieved by SFs with 2 and 3 Å as cutoffs is shown in Table 2, and, notably, IGModel outperforms other SFs by a significant margin. This effectively confirms the generalization ability and robustness of IGModel in different scenarios. However, for CASF2016-AF2 and unbias-v2019-AF2, two datasets based on structures predicted by AlphaFold2, the overall top 1 success rate is lower due to the larger RMSDs of the poses generated by molecular docking relative to the native poses.

**Table 1:** Pearson correlation coefficient (PCC) and Spearman correlation coefficient (SCC) of IGModel and other SFs tested on the CASF2016-AF2, unbias-v2019 and unbias-v2019-AF2 datasets

	CASF2016-AF2		unbias-v2019		unbias-v2019-AF2	
SFs	PCC	SCC	PCC	SCC	PCC	SCC
Vina^a^	0.035	0.071	0.364	0.356	0.209	0.192
DeepBSP^a^	0.401	0.375	0.543	0.507	0.451	0.418
DeepRMSD^a^	0.463	0.430	0.285	0.246	0.261	0.247
DeepRMSD+Vina^a^	0.248	0.290	0.405	0.362	0.299	0.287
GT${}\underline {\ \ }$ft${}\underline {\ \ }$1.0	0.503	0.455				
GatedGCN${}\underline {\ \ }$ft${}\underline {\ \ }$1.0	0.560	0.524				
zPoseScore^a^	0.659	0.593	0.604	0.535	0.554	0.507
IGModel$_{pkd}$	0.736	0.649	0.611	0.522	0.580	0.519
IGModel$_{rmsd}$	0.751	0.669	0.633	0.542	0.609	0.552

^a^The results are cited from reference [46].

**Table 2:** Top1 success rate of IGModel and other SFs tested on the CASF2016-AF2, unbias-v2019 and unbias-v2019-AF2 datasets

	CASF2016-AF2		unbias-v2019		unbias-v2019-AF2	
SFs	2Å	3Å	2Å	3Å	2Å	3Å
Vina^a^	0.127	0.208	0.453	0.526	0.130	0.219
DeepBSP^a^	0.180	0.324	0.453	0.584	0.169	0.254
DeepRMSD^a^	0.269	0.425	0.210	0.324	0.089	0.185
DeepRMSD+Vina^a^	0.261	0.416	0.441	0.534	0.144	0.253
GT${}\underline {\ \ }$ft${}\underline {\ \ }$1.0	0.273	0.448				
GatedGCN${}\underline {\ \ }$ft${}\underline {\ \ }$1.0	0.292	0.465				
zPoseScore^a^	0.339	0.506	0.599	0.700	0.185	0.274
IGModel$_{pkd}$	0.338	0.558	0.562	0.690	0.227	0.318
IGModel$_{rmsd}$	0.364	0.571	0.587	0.690	0.227	0.296

^a^The results are cited from reference [46].

## DISCUSSION

Different SFs have been developed using various training data for pKd or RMSD predictions, and a single score (either a traditional SF or an ML/DL-based SF) may effectively address the quality of docking poses for pose selection [44] and virtual screening [28]. RMSD reflects the difference between the docking pose and the native conformation, providing insight into ‘how it binds’. However, it does not represent the binding strength with the target protein. On the other hand, pKd represents the binding strength between the molecule and the target, addressing the ”how strong it binds’ problem, but it does not reflect the difference between the docking pose and the native pose [66].

Early ML- or DL-based SFs often directly predict protein–ligand binding affinity (pKd) using native protein–ligand complex structures [25, 34–36]. However, testing has shown that the scores of such models are challenging to distinguish correct binding poses generated by docking tools [28], and their accuracies in screening tasks are not satisfactory [37]. Subsequently, researchers began to directly predict the RMSD of docking poses (such as Gnina [67], DeepRMSD [39] and DeepBSP [38]), or use scores from other mathematical spaces (such as DeepDock [40], RTMScore [41] and GenScore [43]) trained with docking poses. The distance likelihood potential generated by DeepDock, RTMScore and Genscore, it is difficult to intuitively describe RMSD and the binding strength and could not provide an explicit and physical meaningful prediction for computational chemists. However, RMSD-corrected pKd is also used as a training target to construct ML models, which provides a direct estimate for both the pose binding pattern and the molecule’s binding strength with a single score [45, 68]. Some representative SFs are listed in Table S8.

In our work, we aim to address both questions of RMSD prediction for ‘how it binds’ and pKd prediction for ‘how strong it binds’ within an integrated framework. We achieve this by characterizing the protein–ligand interaction using two geometric graph modules: the pocket graph module and the protein–ligand atomic graph module. We then apply EdgeGAT layers [50] to encode the interaction features. Tests under different scenarios have demonstrated that IGModel performs well in predicting the RMSD of docking poses for redocking, cross-docking, and AF2-based docking tasks, indicating its excellent generalization ability and robustness for pose selection.

**Figure 6:**
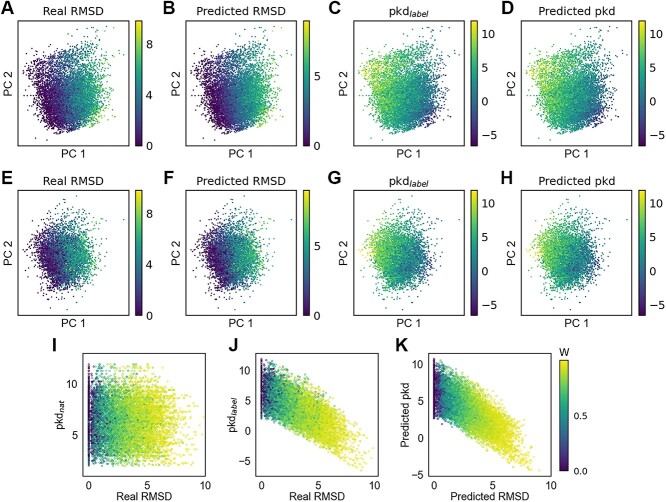
Visualization of the complex embedding, ligand embedding and the decay factor W. (**A**)–(**D**) (**E**–**H**) show the visualization of the complex embedding (ligand embedding) of samples in the validation set after PCA clustering, colored according to real RMSD, predicted RMSD, pKd$_{label}$ and predicted pKd, respectively. (**I**)–(**K**) present visualization of the decay weight of binding strength with respect to RMSD variations, organized according to pKd$_{nat}$-real RMSD, pKd$_{label}$-real RMSD and predicted pKd-Predicted RMSD, respectively, where pKd$_{nat}$ refers to the binding affinity of the native protein–ligand complex.

To visualize the protein–ligand interaction potential energy surface encoded by the graph neural network, we visualize the ligand embedding output by the last EdgeGAT layer in the protein–ligand graph branch, as well as the overall complex embedding. We have employed principal component analysis (PCA) to cluster the latent space and ligand embeddings of samples from the validation set, subsequently coloring them according to actual RMSD, predicted RMSD, pKd$_{label}$ and predicted pKd. The resulting distributions of RMSD and pKd within the latent space are presented in Figure 6(A–D). These plots reveal that as RMSD and pKd vary, distinct stratification patterns become apparent. Analogous patterns are discernible in the ligand embedding plots (Figure 6E–H). A noteworthy observation is the broader coverage area in the latent space images compared to those of ligand embedding, suggesting a superior capacity of the latent space to differentiate between various clusters. This enhanced differentiation can likely be ascribed to the incorporation of protein pocket information into the latent space, thereby refining the depiction of protein–ligand interactions.

IGModel, as the first DL model capable of simultaneously predicting the RMSD of ligand docking poses relative to the native conformation and the binding strength to the target, is based on the assumption of a negative correlation between binding strength and RMSDs of docking poses. The relationship between the decay weight of binding strength and the RMSDs of docking poses remains uncertain. Past research has involved empirically setting functions to model the change in binding strength with RMSD [45, 68], but such manual interventions may introduce biases. We aim for the model to deduce appropriate decay weights autonomously for various protein–ligand complexes, as depicted in Figure 1. The decay weights are represented with respect to pKd$_{nat}$-real RMSD (Figure 6I), pKd$_{label}$-real RMSD (Figure 6J) and predicted pKd-predicted RMSD (Figure 6K). A notable trend is that the decay weight *W* tends to increase with the rise in RMSD, confirming our initial hypothesis that poses closer to the native structure exhibit stronger binding.

Moreover, we investigate the model’s proficiency in learning the physical aspects of protein–ligand interactions. It is widely accepted that protein–ligand binding predominantly involves non-bonded interactions [69], solvation [70] and entropic effects [71]. Specifically, short-range non-bonded interactions like hydrophobic contacts, cation-$\pi $, salt bridges, hydrogen bonds and $\pi $-$\pi $ stacking are known to contribute favorably to the binding free energies of the complexes [69, 72, 73]. However, most current ML/DL-based SFs fail to explicitly or implicitly emphasize or highlight the direct non-bonded interactions between the protein and the ligand.

**Figure 7:**
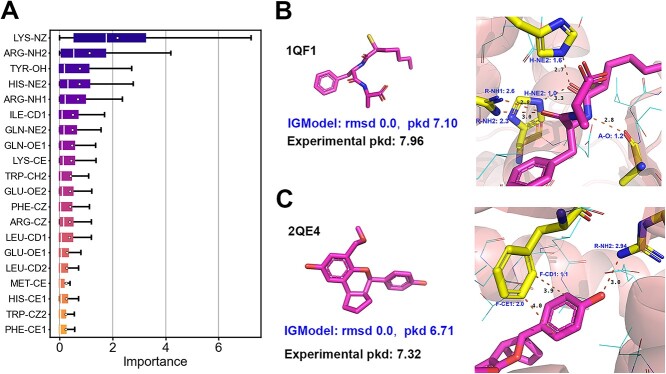
Explainability and case study. (**A**) Ranking of importance of protein atoms at the binding pocket (only the top 20 protein atom types are shown). (**B**) and (**C**) show two cases (1QF1 and 2QE4) where IGModel identifies key interactions, including hydrogen bonding and $\pi $-$\pi $ stacking. In the two pictures located on the right, the distances between key atoms are shown (black), and the names and importance values of key atoms in the protein are colored in blue.

Considering atoms in different residue side chains and even those within the same residue display diverse physicochemical characteristics, it is crucial to account for various features such as residue type, element type, as well as polarity and aromaticity. This approach provides meaningful descriptors for protein atoms, enabling IGModel to identify essential non-bonded interactions like hydrogen bonds, as illustrated in Figure 7. We analyzed the attention values assigned by IGModel to each protein–ligand atom pair in the protein–ligand complexes from the CASF-2016 dataset to determine if the model effectively highlights the physical interactions. A protein atom’s importance is determined by the aggregated attention values from the interaction edges with the ligand atoms. We compiled the importance scores for all types of protein atoms and presented the top 20 in Figure 7. It is evident that among the most critical protein atoms, polar atoms are predominant, underscoring their vital role of polar interactions in protein–ligand binding [72] for binding strength and binding specificity. Moreover, certain non-polar atoms, such as ILE-CD1 and PHE-CZ, are also identified as significant contributors. This significance arises as alpha carbon atoms and aromatic rings are commonly involved in hydrophobic interactions, among the most prevalent in protein–ligand binding. Figure 7(B) illustrates that protein atoms engaged in hydrogen bonding receive higher importance scores, indicating that IGModel effectively recognizes hydrogen bonds and allocates increased attention to them. In Figure 7(C), the importance value is notably high for the edges of the benzene ring in phenylalanine when proximal to the ligand’s benzene ring, suggesting that IGModel can aptly detect $\pi $-$\pi $ stacking interactions.

## CONCLUSION

In this study, we introduce IGModel, a novel scoring framework for predicting protein–ligand interactions, capable of concurrently predicting RMSD of ligand binding poses and binding strength to the target. The model employs the EdgeGAT layers to encode input graphs into a latent space that characterizes protein–ligand interactions, subsequently decoding this space into RMSD and pKd values using two separate decoders. IGModel has demonstrated SOTA performance across various docking power test sets. While designed to provide a holistic assessment of docking poses, IGModel also excels in scoring power and ranking power tests on the CASF-2016 benchmark, matching or surpassing other models. Additionally, it outperforms most baseline SFs in screening power. The robustness of IGModel is further validated on the challenging unbias-v2019 set and datasets containing targets predicted by AlphaFold2, showcasing its strong generalizability. Visualization of the latent space encoded by IGModel offers an intuitive depiction of the energy landscape that describes the RMSD and binding strength of docking poses. Case studies reveal IGModel’s adeptness in identifying key interactions like hydrogen bonding and $\pi $-$\pi $ stacking.

It is acknowledged that no single SF can excel in all tasks, yet IGModel manages to achieve relatively balanced performance. Significantly, IGModel pioneers a novel approach in predicting protein–ligand interactions, moving beyond the traditional single-score output of SFs, to ensure outputs with clear physical interpretation. We posit that this framework holds substantial promise for molecular docking applications and the refinement and optimization of lead compounds in drug design. In conclusion, our research presents a new direction for the future design of SFs and opens up fresh challenges in the field.

**Key Points**

- We introduce the first framework for simultaneously predicting the RMSD of the ligand docking pose and its binding strength to the target.
- IGModel can effectively improve the accuracy of identifying the near-native binding poses of the ligands, and can still outperform most baseline models in scoring power, ranking power and screening power tasks.
- IGModel is still ahead of other state-of-the-art models in the unbiased data set and the target structure predicted by AlphaFold2, proving its excellent generalization ability.
- Latent space provided by IGModel learns the physical interactions, thus indicating the robustness of the model.

## Supplementary Material

revised_SI_bbae145

## Author Biographies

**Zechen Wang** is a PhD candidate student at the School of Physics, Shandong University, China. He is working on protein-ligand interaction prediction and drug design.

**Sheng Wang** is the CEO (Chief Executive Officer) of Shanghai Zelixir Biotech Company Ltd. His research area is protein structure prediction and AI-based synthetic biology.

**Yangyang Li** is currently a professor at the School of Physics, Shandong University. His research interests focus on the investigation of novel physical (quantum) properties and functionalities of transition-metal-oxides with strongly correlated electrons.

**Jingjing Guo** is a professor at the Centre in Artificial Intelligence Driven Drug Discovery, Faculty of Applied Science, Macao Polytechnic University. Her research interests are computational biology and AI-drug discovery.

**Yanjie Wei** is the executive director of Center for High Performance Computing at Shenzhen Institutes of Advanced Technology, Chinese Academy of Sciences. His research area is bioinformatics and high performance computing.

**Yuguang Mu** is an associate professor at the School of Biological Sciences, Nanyang Technological University, Singapore. He is working on protein dynamics, molecular docking and drug discovery.

**Liangzhen Zhen** is a senior research scientist at Shanghai Zelixir Biotech Company Ltd. He is also an Associate Investigator at Shenzhen Institute of Advanced Technology, Chinese Academy of Sciences. He is working on protein modeling and protein-ligand interaction modeling.

**Weifeng Li** is a full professor at the School of Physics, Shandong University. He is interested in multi-scale simulations of biological systems and drug development.
