# Correction to: A new paradigm for applying deep learning to protein–ligand interaction prediction

**DOI:** 10.1093/bib/bbae226

**Published:** 2024-05-03

This is a correction to:

Zechen Wang, Sheng Wang, Yangyang Li, Jingjing Guo, Yanjie Wei, Yuguang Mu, Liangzhen Zheng, Weifeng Li, A new paradigm for applying deep learning to protein–ligand interaction prediction, *Briefings in Bioinformatics*, Volume 25, Issue 3, May 2024, bbae145, https://doi.org/10.1093/bib/bbae145

In the originally published version of this manuscript, there are four inaccuracies in the data descriptions within the main text, which exhibit minor discrepancies compared to the data in Table S3. Additionally, there are compilation errors with certain symbols (underlines) in the published PDF version. Finally, the corresponding authors' address is not shown in the PDF version.

These errors have been corrected.

