# Supplementary material for: A new paradigm for applying deep learning to protein–ligand interaction prediction
Source: Brief Bioinform. 2024 Apr 5;25(3):bbae145. doi: 10.1093/bib/bbae145 (PMC10998640; doi:10.1093/bib/bbae145)
Supplement: revised_SI_bbae145 [file revised_si_bbae145.docx]

**Supporting Materials**

**Part 1. Details of docking poses in the training set.**

In this work, docking poses were generated by AutoDock Vina[1] and Ledock[2], where the search space is a 15Å*15Å*15Å cubic, the maximum number generated is set to 10, and other parameters are used by default. The RMSD distribution of docking poses and the pKd distribution of the native conformation corresponding to these docking poses are shown in Figure S1.


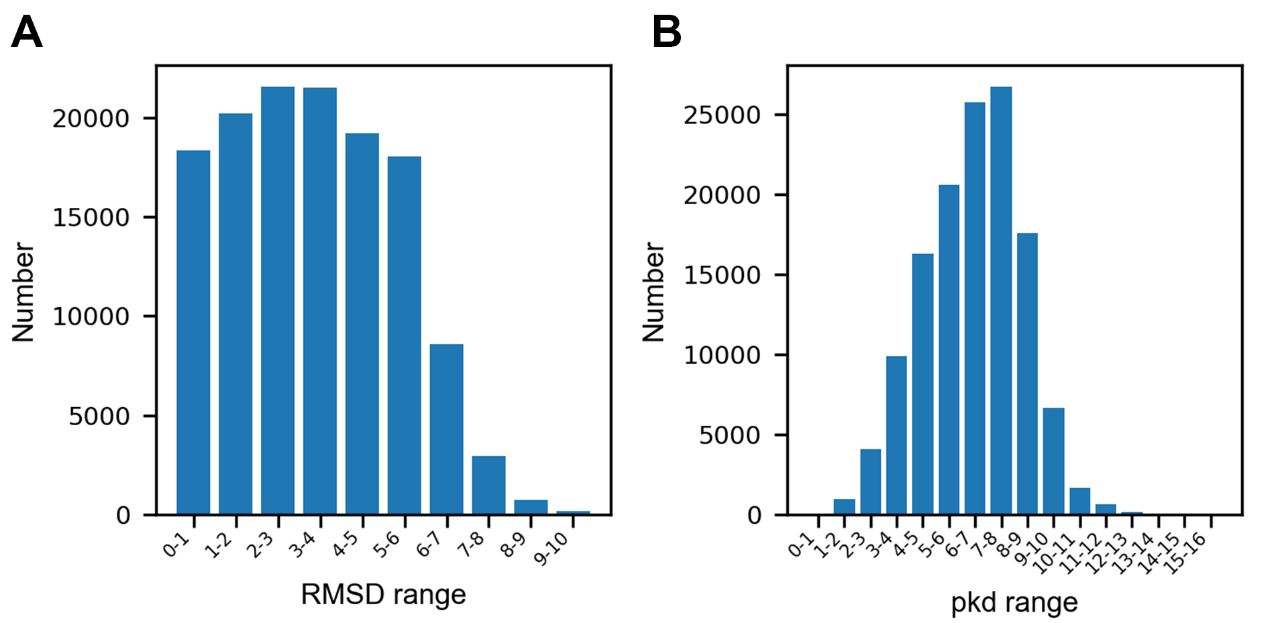


**Figure S1.** The RMSD (**A**) and native pKd (**B**) distributions of docking poses in the training set.

**Part 2. Details of node features and edge features of graphs**

**Table S1.** Node and Edge Features employed for protein-ligand interaction graph

| Features | | Size |
| --- | --- | --- |
| Nodes (Ligand atoms) | | |
| One hot encoding for the element of the atom (“C”, “N”, “O”, “P”, “S”, “Hal”, “DU”) | | 7 |
| Edges (Ligand-Ligand nodes) | | |
| One hot encoding for the bond type (“SINGLE”, “DOUBLE”, “TRIPLE”, “AROMATIC”) | | 4 |
| Whether the bond is conjugated | | 1 |
| Whether the bond is in ring | | 1 |
| One hot encoding for the stereo configuration the bond (“STEREONONE”, “STEREOANY”, “STEREOZ”, “STEREOE”) | | 4 |
| The edge length | | 1 |
| Nodes (Protein atoms) | | |
| One hot encoding for residue type (“GLY”, “ALA”, “VAL”, “LEU”, “ILE”, “PRO”, “PHE”, “TYR”, “TRP”, “SER”, “THR”, “CYS”, “MET”, “ASN”, “GLN”, “ASP”, “GLU”, “LYS”, “ARG”, “HIS”, “OTH”) | | 21 |
| One hot encoding for the element of the atom (“C”, “N”, “O”, “S”, “DU”) | | 5 |
| Whether the atom is in the main chain ([1, 0] or [0, 1]) | | 2 |
| Whether the atom is aromatic ([1, 0] or [0, 1]) | | 2 |
| One hot encoding for charge (-1, 0, 1) of the atom | | 3 |
| Distance between the atom and the α-C atom | | 1 |
| Edges (Protein-Protein nodes) | | |
| The edge length |  | 1 |
| Edges (Protein-Ligand nodes) | | |
| The edge length | | 1 |
| sin(φ/2), φ is the dihedral angle formed by the ligand center, the ligand atom, the protein atom and the α-C atom of the residue where the protein atom is located | | 1 |
| cos(θ1 ), θ1 is the angle formed by the ligand atom, the protein atom and the α-C atom of the residue where the protein atom is located | | 1 |
| cos(θ2 ), θ2 is the angle formed by the protein atom, the ligand atom and the ligand center | | 1 |

**Table S2.** Node and Edge Features Employed for protein pocket graph

| Features | Size |
| --- | --- |
| Nodes |  |
| One hot encoding for residue type (“GLY”, “ALA”, “VAL”, “LEU”, “ILE”, “PRO”,“PHE”, “TYR”, “TRP”, “SER”, “THR”, “CYS”, “MET”, “ASN”, “GLN”, “ASP”, “GLU”, “LYS”, “ARG”, “HIS”, “OTH”) | 21 |
| Max distance between any atom and α-C atom | 1 |
| Distance between atoms named C and N | 1 |
| Distance between α-C atom and the pocket center | 1 |
| Max and Min distance between any atom and the pocket center | 2 |
| sin(ϕ/2) and sin(ψ/2), where ϕ and ψ are main chain dihedral angles | 2 |
| sin(χi /2), where χi (i = 1, 2, 3, 4 and 5) is the side chain dihedral angle. If a diheral angle does not exist in the residue, it is set to -2. | 5 |
| Edges |  |
| Distance between the α-C atoms of two residues | 1 |
| Distance between the main-chain carboxyl O atoms of two residues | 1 |
| Distance between the main-chain N atoms of two residues | 1 |
| Distance between the main-chain carboxyl C atoms of two residues | 1 |
| Distance between the centers of two residues | 1 |
| Max and min distance between two residues | 2 |

**Part 3. Performance of IGModel and some representative scoring functions in CASF-2016 benchmark**

**
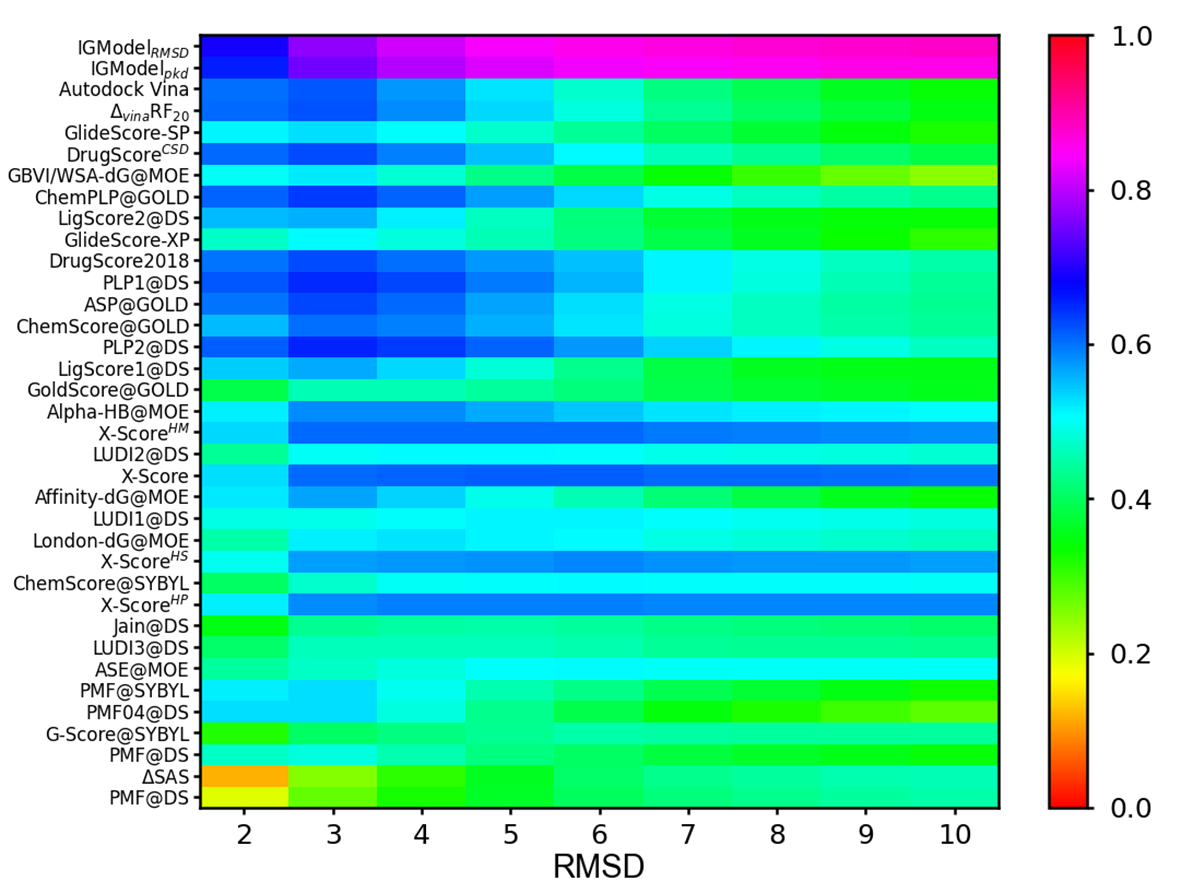
**

**Figure S2.** The IGModel achieved average Spearman correlation coefficient (SCC) on the decoy set of 285 protein-ligand pairs, categorized by different RMSD intervals. The x-axis indicates the RMSD range (e.g., [0-2Å], [0-3Å], and so on). Within these nine RMSD ranges, the average SCCs achieved by IGModel_rmsd_ are 0.688, 0.773, 0.816, 0.842, 0.857, 0.865, 0.872, 0.876, and 0.880, while those achieved by IGModel_pkd_ are 0.658, 0.750, 0.793, 0.821, 0.838, 0.846, 0.855, 0.860, and 0.863. Compared to the scoring functions reported in the CASF-2016 literature[3], it can be seen that IGModel has an obviously advantage.

**Table S3.** The docking power and screening power of several representative SFs on the CASF-2016 benchmark.

| Scoring function | docking power | | screening power | |
| --- | --- | --- | --- | --- |
|  | top 1 success rate (w/o native poses) | top 1 success rate (with native poses) | top 1% success rate | enrichment factor (1%) |
| AutoDock Vina | 0.846 | 0.902 | 0.298 | 7.70 |
| ChemPLP@GOLD | 0.832 | 0.860 | 0.351 | 11.91 |
| GlideScore-SP | 0.846 | 0.877 | 0.368 | 11.44 |
| ∆_Vina_RF_20_ | 0.849 | 0.891 | 0.421 (0.456) | 11.73 (12.36) |
| ∆_Vina_ XGB |  | 0.920 | 0.368 | 13.14 |
| ∆_LinF9_ XGB |  | 0.867 | 0.404 | 12.61 |
| OnionNet-SFCT+Vina |  | 0.937 | 0.421 | 15.50 |
| DeepBSP | 0.872 | 0.885 |  |  |
| DeepDock |  | 0.870 | 0.439 | 16.41 |
| DeepRMSD+Vina | 0.916 | 0.944 | 0.474 | 21.95 |
| RTMScore | 0.934 | 0.973 | 0.667 | 28.0 |
| GT_ft_1.0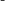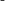 | 0.940 | 0.966 | 0.719 | 28.12 |
| GatedGCN_ft_1.0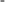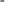 | 0.926 | 0.954 | 0.661 | 23.54 |
| IGModel_self-ref_pkd_ | 0.775 | 0.800 |  |  |
| IGModel_self-ref_rmsd_ | 0.888 | 0.919 |  |  |
| IGModel_pkd_* | 0.909 | 0.933 | 0.667 | 19.40 |
| IGModel_rmsd_ | 0.951 | 0.975 |  |  |

Note: The results of SFs other than IGModel are cited from the references.

*In the CASF-2016 screening power test, IGModel_pkd_ achieved success rates of 66.7%, 84.2% and 87.7% at the Top 1%, Top 5%, and Top10% levels, respectively. The enrichment factors for Top 1%, Top 5% and Top 10% were 19.4, 6.87, and 4.33.

**Table S4.** The comparison of IGModel and several representative SFs in the scoring power and ranking power tests on CASF-2016.

|  |  | Scoring power | | Ranking power |  |
| --- | --- | --- | --- | --- | --- |
| Year | SFs | PCC | RMSE | Spearman correlation coefficient | Training set |
| 2023 | IGModel_pkd_* | 0.831 | 1.254 | 0.723 | PDBbind v2019 (general set) |
| 2023 | GatedGCN_ft_1.0 | 0.834 |  | 0.686 | PDBbind v2020 (general set) |
| 2023 | GT_ft_1.0 | 0.802 |  | 0.684 | PDBbind v2020 (general set) |
| 2023 | PLANET | 0.824 | 1.247 | 0.682 | PDBbind v2020 (general set) |
| 2022 | RTMScore | 0.455 |  | 0.529 | PDBbind v2020 (general set) |
| 2022 | MPNN | 0.813 | 1.511 |  | PDBbind v2016 (general set) |

Note: The results of SFs other than IGModel are cited from the references.

*For the scoring power, the standard deviation (SD) between the predicted pKd values by IGModel_pkd_ and the experimental pKd values is 1.21. For the ranking power, the Kendall correlation coefficient and Predictive index (PI) are 0.635 and 0.749, respectively.

The Pearson correlation coefficient and RMSE achieved by IGModel_pkd_ on CASF-2016 core set are 0.831 and 1.254 respectively, as shown in Figure S2A. Figure S2B shows the result of IGModel_pkd_ on the validation set, including crystal structures and decoys.

**
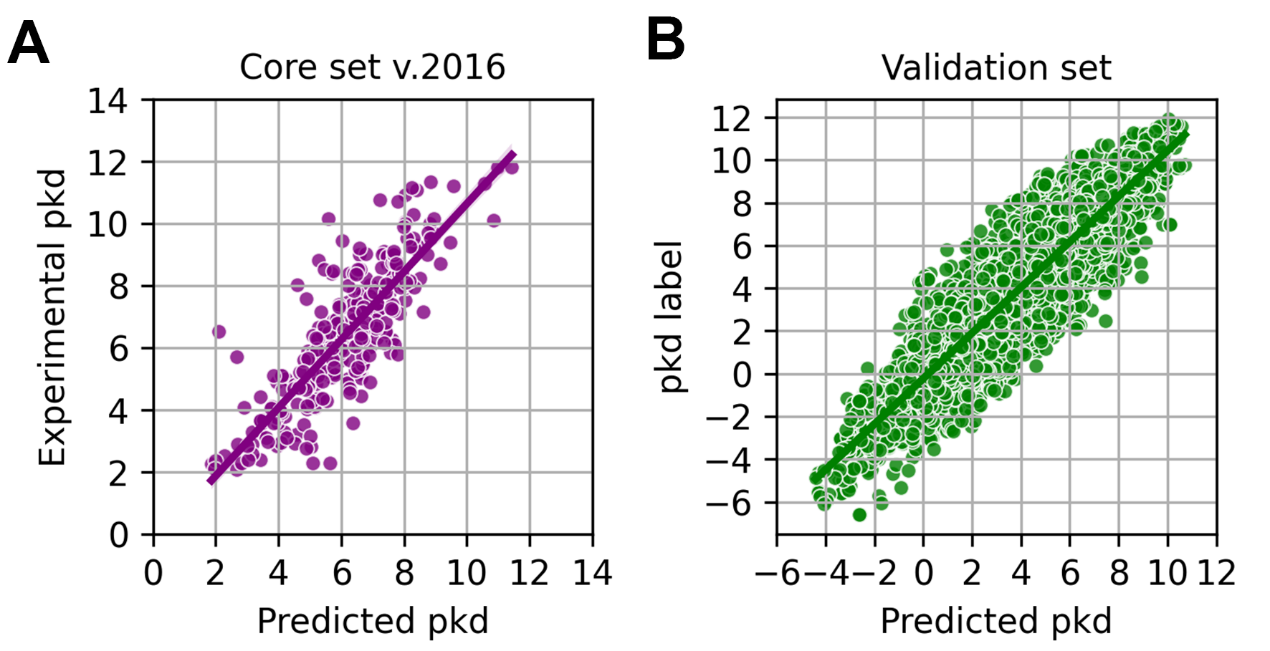
**

**Figure S3.** **A.** The correlation between the pKd predicted by IGModel_pkd_ and the experimental pKd for 285 native protein-ligand complexes in the CASF-2016 test set. **B.** The scatter plot of pKd predicted by IGModel_pkd_ against the labels on the validation set.

**Part 4. Ablation experiments**

As shown in Table S5, the removal of the protein atoms’ location feature (within the main/side chain) , Angle 1, Angle 2, or the dihedral angle formed between the protein and the ligand in the protein-ligand atomic interaction graph, as well as the removal of the pocket graph, resulted in varying degrees of performance decrease for IGModel.

**Table S5.** The ablation experiment of IGModel on the validation set and CASF-2016 docking power

|  | Valid | | | | CASF-2016 | | | |
| --- | --- | --- | --- | --- | --- | --- | --- | --- |
|  | RMSD | | pKd | | RMSD (Top 1 success rate) | | pKd (Top 1 success rate) | |
|  | PCC | RMSE | PCC | RMSE | including crystal poses | excluding crystal poses | including crystal poses | excluding crystal poses |
| IGModel W/O protein atoms’ location feature | 0.918 | 0.828 | 0.892 | 1.193 | 96.5% | 94.4% | 83.9% | 82.5% |
| IGModel W/O Angle_1 | 0.917 | 0.815 | 0.896 | 1.216 | 96.1% | 94.0% | 92.3% | 88.4% |
| IGModel W/O Angle_2 | 0.923 | 0.787 | 0.902 | 1.247 | 97.9% | 94.4% | 93.7% | 90.5% |
| IGModel W/O cplx dihedral | 0.923 | 0.792 | 0.898 | 1.276 | 95.1% | 91.9% | 92.3% | 87.4% |
| IGModel W/O pocket graph | 0.894 | 0.914 | 0.895 | 1.281 | 94.7% | 91.2% | 86.3% | 81.4% |
| IGModel | 0.927 | 0.768 | 0.905 | 1.220 | 97.5% | 95.1% | 93.3% | 90.9% |

**Part 5. Performance of IGModel on ligand binding poses generated by DiffDock**

To evaluate the performance of IGModel on poses generated by AI-based methods, we created a small dataset following these steps: Initially, we regenerated complexes for 285 protein-ligand pairs from the CASF-2016 test set using DiffDock with default parameters. Each protein-ligand pair was configured to generate 40 binding poses. Subsequently, to ensure that all generated structures resided within the binding pocket, poses with an RMSD greater than 10Å were excluded, resulting in a total of 9777 binding poses across all 285 protein-ligand pairs. We utilized IGModel to predict the RMSD for these binding poses, achieving a Pearson correlation coefficient (PCC) of 0.807, a Spearman correlation coefficient (SCC) of 0.755 and an RMSE of 0.875 against the actual RMSD. The scatter plot is presented in Figure S4. Then we calculated the average PCC and SCC of IGModel_rmsd_ and IGModel_pkd_ on the decoy sets of 285 protein-ligand pairs, and compared them with GatedGCN_ft_1.0, GT_ft_1.0 and Vina score. The results are presented in the Table S6. Since the CASF-2016 test set is included in the DiffDock training set, it is easier to generate a large number of low RMSD binding poses. As shown in the Table S6, the SCC achieved by several scoring functions are all around 0.55, indicating that they can still achieve normal performance on the structure generated by DiffDock.

**
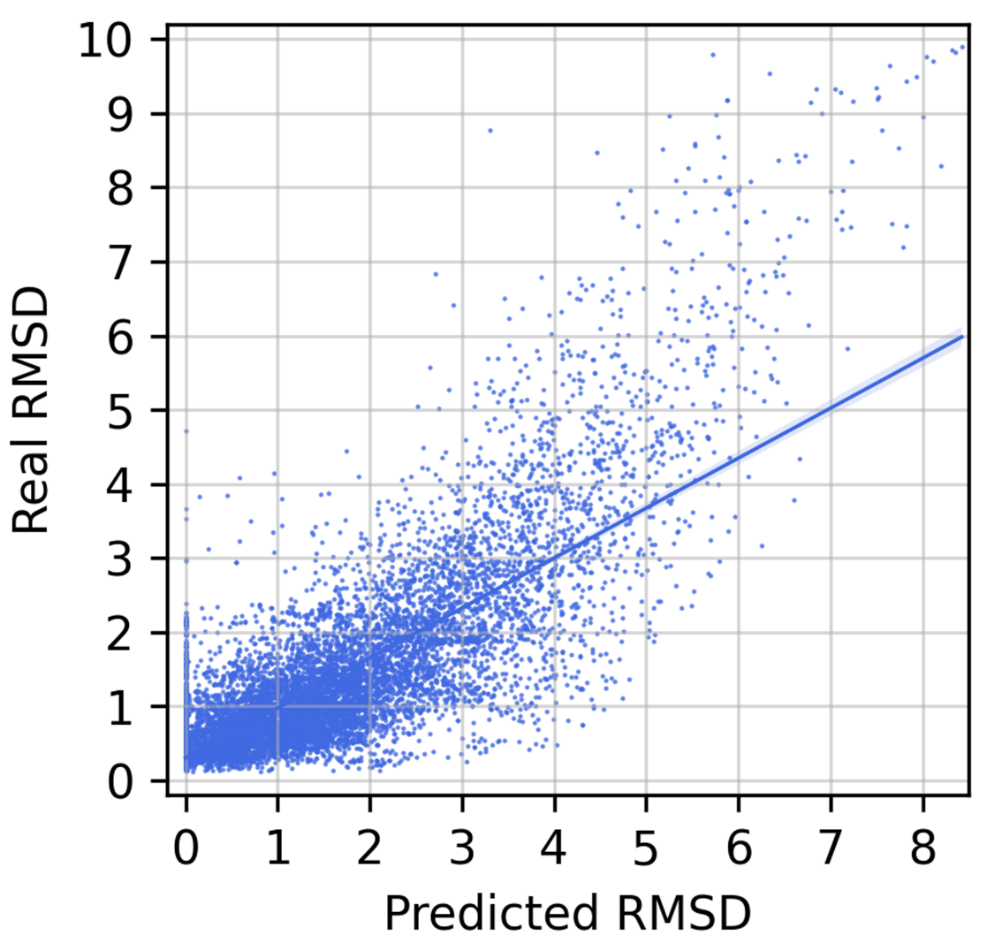
**

**Figure S4.** The scatter plot of IGModel_rmsd_ on binding poses generated by DiffDock.

**Table S6.** The average PCC and SCC achieved by IGModel on the decoy sets generated by DiffDock for 285 protein-ligand paits.

| SFs | Ave. PCC | Ave. SCC |
| --- | --- | --- |
| IGModel_RMSD_ | 0.672 | 0.574 |
| IGModel_pkd_ | 0.653 | 0.546 |
| GatedGCN_ft_1.0 | 0.627 | 0.540 |
| GT_ft_1.0 | 0.629 | 0.539 |
| Vina score | 0.561 | 0.543 |

**Part 6. Performance of IGModel on DUD-E and DUD-AD.**

DUD-E is a commonly used benchmark to evaluate the screening ability of scoring functions. It includes 102 targets, and each target contains an average of 224 active molecules and 11,200 decoy molecules[4]. Later, Chen et al.[5] proposed the DUD-AD set in order to hide the bias in DUD-E. Specifically, for each target, the active molecules of other targets are used as decoys. Here, the tested DUD-E poses are sourced from the open-access database provided by Shen et al.[6] The docking poses in DUD-AD were generated using AutoDock Vina, with the search space defined as a cubic box of 15Å*15Å*15Å[7].

The performance of IGModel and several representative SFs on these two test sets is presented in Figure S5 and Table S7. Whether on DUD-E or DUD-AD sets, RTMScore achieved the highest EF (0.5%, 1.0% and 5.0%), followed by GenScore (namely GatedGCN_ft_1.0 and GT_ft_1.0). Although the AUC values achieved by IGModel_pkd_ and IGModel_self-ref_pkd_ on the unbiased DUD-AD set (0.686 and 0.692) are slightly higher than those achieved by RTMscore (0.658), GatedGCN_ft_1.0 (0.647), and GT_ft_1.0 (0.661), respectively, EF more directly reflects the performance of SFs in actual virtual screening. It can be clearly seen that in the screening task, there is still a gap between IGModel and SOTA SFs.

**Table S7.** Comparison of SFs on the DUD-AD and DUD-E sets according to the average EF (0.5%, 1% and 5%) and average AUROC.

|  | DUD-AD | | | | DUD-E | | | |
| --- | --- | --- | --- | --- | --- | --- | --- | --- |
|  | EF (0.5%) | EF (1%) | EF (5%) | AUC | EF (0.5%) | EF (1%) | EF (5%) | AUC |
| Vina Score | 2.30 | 1.92 | 1.35 | 0.477 | 12.06 | 9.76 | 4.79 | 0.711 |
| DeepRMSD+Vina | 7.01 | 6.04 | 3.40 | 0.591 | 26.50 | 21.03 | 8.66 | 0.806 |
| RTMScore* | 12.99 | 10.65 | 5.10 | 0.658 | 42.47 | 35.10 | 10.88 | 0.830 |
| GatedGCN_ft_1.0* | 12.19 | 9.77 | 4.52 | 0.647 | 38.91 | 31.21 | 10.56 | 0.824 |
| GT_ft_1.0* | 10.96 | 9.48 | 4.89 | 0.661 | 40.56 | 32.65 | 10.66 | 0.824 |
| IGModel_pkd_ | 10.10 | 8.73 | 4.89 | 0.686 | 27.98 | 22.15 | 8.31 | 0.782 |
| IGModel_self-ref_pkd_ | 8.82 | 7.74 | 4.52 | 0.692 | 27.56 | 22.57 | 8.77 | 0.799 |

Note: *The EF and AUC values on the DUD-E set are sourced from the literature[6, 8].

**Part 7. Description of some representative scoring functions**

**Table S8. Information of some representative scoring functions**

| **Scoring function (SF)** | **Classification** | **Description** | **Reference** |
| --- | --- | --- | --- |
| AutoDock Vina | Empirical SF | Comprised of five energy terms manually tailored. | [1] |
| X-Score | Empirical SF | Comprised of manually configured energy terms, including van der Waals interactions, hydrogen bonding, deformation effects, and hydrophobic interactions. | [9] |
| ChemPLP@GOLD | Empirical SF | Comprised of manually configured energy terms, including ligand clash potential, ligand torsional potential, hydrogen bonding, and metal interactions. | [10] |
| GlideScore-SP | Empirical SF | Composed of manually configured energy terms, including lipophilic-lipophilic term, hydrogen bonding, metal-ligand interactions, Coulomb interactions, van der Waals interactions, solvent terms, and so on | [11, 12] |
| DrugScore2018 | Knowledge-based SF | scoring protein-ligand interactions through a distance-dependent potential between protein and ligand atoms | [13] |
| ∆_Vina_RF_20_ | ML-based SF | employ random forest to parameterize corrections to the AutoDock Vina scoring function | [14] |
| ∆_Vina_ XGB | ML-based SF | employ eXreme Gradient Boosting (XGBoost) to parameterize corrections to the AutoDock Vina scoring function | [15] |
| ∆_LinF9_ XGB | ML-based SF | employ eXreme Gradient Boosting (XGBoost) to parameterize corrections to machine learning scores (Lin_F9) | [16] |
| OnionNet-SFCT+Vina | Integrating empirical SF and ML model | ML score is employed as an correction term to the Vina score, enhancing both docking power and screening power. | [7] |
| DeepBSP | DL-based SF | Modeling protein-ligand complexes using three-dimensional voxel grids, followed by applying a 3D CNN to predict the RMSD of ligand binding poses | [17] |
| DeepDock | DL-based SF | Utilizing a graph neural network to learn the distance likelihood between protein and ligand, and subsequently deriving the potential of the protein-ligand complex based on this learned distance likelihood. | [18] |
| DeepRMSD+Vina | Integrating empirical SF and DL model | Integrating the DL-based RMSD prediction model with Vina score to enhance both docking power and screening power | [19] |
| RTMScore | DL-based SF | Utilizing a graph transformer model to learn the distance likelihood between protein and ligand, and subsequently deriving the potential of the protein-ligand complex based on this learned distance likelihood. | [6] |
| PLANET | DL-based SF | Applying Graph Neural Networks to model protein-ligand complexes for predicting binding affinity. | [20] |
| GenScore | DL-based SF | The modeling of protein-ligand complexes follows a strategy similar to RTMScore. Fine-tuning of the foundational model is conducted with affinity data, resulting in state-of-the-art performance across scoring, ranking, docking, and screening tasks | [8] |
| IGModel | DL-based SF | Applying graph neural networks to model protein-ligand complexes, simultaneously predicting the RMSD of ligand binding poses and the binding affinity with the target. |  |

**Reference**

1. Trott O, Olson AJ. AutoDock Vina: improving the speed and accuracy of docking with a new scoring function, efficient optimization, and multithreading, J Comput Chem 2010;31:455-461.

2. Liu N, Xu Z. Using LeDock as a docking tool for computational drug design, IOP Conference Series: Earth and Environmental Science 2019;218.

3. Su M, Yang Q, Du Y et al. Comparative Assessment of Scoring Functions: The CASF-2016 Update, J Chem Inf Model 2019;59:895-913.

4. Mysinger MM, Carchia M, Irwin JJ et al. Directory of Useful Decoys, Enhanced (DUD-E): Better Ligands and Decoys for Better Benchmarking, Journal of Medicinal Chemistry 2012;55:6582-6594.

5. Chen L, Cruz A, Ramsey S et al. Hidden bias in the DUD-E dataset leads to misleading performance of deep learning in structure-based virtual screening, PLoS One 2019;14:e0220113.

6. Shen C, Zhang X, Deng Y et al. Boosting Protein-Ligand Binding Pose Prediction and Virtual Screening Based on Residue-Atom Distance Likelihood Potential and Graph Transformer, J Med Chem 2022;65:10691-10706.

7. Zheng L, Meng J, Jiang K et al. Improving protein-ligand docking and screening accuracies by incorporating a scoring function correction term, Brief Bioinform 2022;23:bbac051.

8. Shen C, Zhang X, Hsieh CY et al. A generalized protein-ligand scoring framework with balanced scoring, docking, ranking and screening powers, Chem Sci 2023;14:8129-8146.

9. Wang R, Lai L, Wang S. Further development and validation of empirical scoring functions for structure-based binding affinity prediction, J Comput Aided Mol Des 2002;16:11-26.

10. Korb O, Stutzle T, Exner TE. Empirical scoring functions for advanced protein− ligand docking with PLANTS, Journal of Chemical Information and Modeling 2009;49:84-96.

11. Friesner RA, Banks JL, Murphy RB et al. Glide: a new approach for rapid, accurate docking and scoring. 1. Method and assessment of docking accuracy, Journal of Medicinal Chemistry 2004;47:1739-1749.

12. Halgren TA, Murphy RB, Friesner RA et al. Glide: a new approach for rapid, accurate docking and scoring. 2. Enrichment factors in database screening, Journal of Medicinal Chemistry 2004;47:1750-1759.

13. Dittrich J, Schmidt D, Pfleger C et al. Converging a Knowledge-Based Scoring Function: DrugScore(2018), J Chem Inf Model 2019;59:509-521.

14. Wang C, Zhang Y. Improving scoring-docking-screening powers of protein-ligand scoring functions using random forest, J Comput Chem 2017;38:169-177.

15. Lu J, Hou X, Wang C et al. Incorporating Explicit Water Molecules and Ligand Conformation Stability in Machine-Learning Scoring Functions, J Chem Inf Model 2019;59:4540-4549.

16. Yang C, Zhang Y. Delta Machine Learning to Improve Scoring-Ranking-Screening Performances of Protein-Ligand Scoring Functions, J Chem Inf Model 2022;62:2696-2712.

17. Bao J, He X, Zhang JZH. DeepBSP-a Machine Learning Method for Accurate Prediction of Protein-Ligand Docking Structures, J Chem Inf Model 2021;61:2231-2240.

18. Méndez-Lucio O, Ahmad M, del Rio-Chanona EA et al. A geometric deep learning approach to predict binding conformations of bioactive molecules, Nat Mach Intell 2021;3:1033-1039.

19. Wang Z, Zheng L, Wang S et al. A fully differentiable ligand pose optimization framework guided by deep learning and a traditional scoring function, Brief Bioinform 2023;24:bbac520.

20. Zhang X, Gao H, Wang H et al. PLANET: A Multi-objective Graph Neural Network Model for Protein-Ligand Binding Affinity Prediction, J Chem Inf Model 2023.
